# Evaluation of perfusion parameters of gingival inflammation using laser Doppler flowmetry and tissue spectrophotometry– a prospective comparative clinical study

**DOI:** 10.1186/s12903-023-03507-9

**Published:** 2023-10-14

**Authors:** Marie Sophie Katz, Mark Ooms, Philipp Winnand, Marius Heitzer, Anna Bock, Kristian Kniha, Frank Hölzle, Ali Modabber

**Affiliations:** Department of Oral and Maxillofacial Surgery, University Hospital RWTH Aachen, Pauwelstraße 30, Aachen, 52074 Germany

**Keywords:** Laser-doppler flowmetry, Tissue spectrophotometry, Gingivitis, Inflammation, Perfusion, Oxygenation, Hyperemia

## Abstract

**Background:**

The aim of this study was to determine the values of different perfusion parameters- such as oxygen saturation, the relative amount of hemoglobin, and blood flow- in healthy subjects compared to patients with gingivitis as a non-invasive measurement method.

**Methods:**

A total of 114 subjects were enrolled in this study and separated into subjects with gingivitis (50) and without gingivitis (64) based on clinical examination. Gingival perfusion was measured at 22 points in the maxilla and mandible using laser Doppler flowmetry and tissue spectrophotometry (LDF-TS) with the “oxygen to see” device. All patients underwent measurement of gingival perfusion, followed by the clinical evaluation (measurement of probing depths, evaluation of bleeding on probing, plaque level, and biotype). Perfusion parameters were compared between the groups, associations between the non-invasive and clinical measurements were analyzed, and theoretical optimal cut-off values for predicting gingivitis were calculated with receiver operating characteristics.

**Results:**

The mean oxygen saturation, mean relative amount of hemoglobin, and mean blood flow all significantly differed between the groups with and without gingivitis (p = 0.005, p < 0.001, and p < 0.001, respectively). The cut-off value for predicting gingivitis was > 40 AU (p < 0.001; sensitivity 0.90, specificity 0.67).

**Conclusions:**

As a non-invasive method, LDF-TS can help determine gingival hyperemia. Flow values above 40 AU indicate a higher risk of hyperemia, which can be associated with inflammation. The LDF-TS method can be used for the objective evaluation of perfusion parameters during routine examinations and can signal the progression of hyperperfusion before any change in clinical parameters is observed.

**Trial registration:**

All procedures performed in this study involving human participants were in accordance with the ethical standards of the institutional and/or national research committee and with the 1964 Helsinki Declaration and its later amendments or comparable ethical standards. The study was approved by the institutional Clinical Research Ethics Committee (Ethik-Kommission der Medizinischen Fakultät der RWTH Aachen, Decision Number 286/20) and retrospectively registered by the German Clinical Trials Register (File Number DRKS00024048, registered on the 15th of October 2021).

**Supplementary Information:**

The online version contains supplementary material available at 10.1186/s12903-023-03507-9.

## Background

Gingivitis, a reversible inflammation of the gum, but the potential precursor of periodontal disease, is a common diagnosis in dental examination [1, 2]. Diagnostics and patient education regarding this condition and its treatment options are important, as further damage to the periodontium can be prevented [3–5]. Like any other inflammatory reaction, gingivitis is associated with increased perfusion of the gum, leading to the immigration of inflammatory cells and mediators [6, 7]. These processes lead to typical clinical signs of inflammation, such as redness, swelling, hyperthermy, and pain [8]. Since gingival bleeding is the main marker of gingival inflammation, several approaches have been developed to detect and grade gingival hyperemia. During clinical examination, bleeding on probing is a reliable parameter of gingival hyperemia, and can be graded using classifications like the Papilla Bleeding Index (PBI) by Saxer and Mühlemann [9]. Furthermore, there are classification systems such as the Gingiva Index (GI) developed by Löe and Silness [10] that also consider the appearance of the gingiva. However, visual assessments are associated with some level of examiner-related subjectivity [11]. Besides that, BOP and the indices evaluating its intensity are also correlated with the pressure applied on the dental probe and have a low sensitivity [12].

Aside from these conventional examination methods, some prior studies have used laser Doppler flowmetry (LDF) as a precise method to determine the amount of gingival hyperemia [13–15]. This non-invasive technique can detect changes in the microcirculation of tissues. While LDF alone can only measure blood flow, the use of LDF in combination with tissue spectrophotometry (LDF-TS) can also identify oxygen saturation SO_2_ (%) and the relative amount of hemoglobin rHb in arbitrary units (AU). This data can provide useful information regarding local perfusion and metabolism.

The combination of LDF-TS, which is used in the “oxygen to see” (O2C) device (LEA-Medizintechnik, Gießen, Germany), is a common tool in maxillofacial and plastic surgery to monitor microvascular transplants [16–19]. Although extraoral use of this tool has become a standardized and secure option, intraoral use is more difficult to establish, as smaller probes are needed, and the placement of probes is less stable. Lacking a specialized measurement tool for intraoral use, in a former study by Barry et al. a probe head of the O2C device was connected to a dental probe to enable intraoral measurements [20].

The present study was the first to use a manufactured gingival probe head connected to the O2C device. This clinical trial aimed to compare various perfusion parameters– such as gingival hemoglobin saturation and blood flow– between healthy subjects and patients with gingivitis using a new, non-invasive measurement method in addition to the conventional clinical examination to improve the diagnosis of gingival inflammation and to use it as a diagnostic tool to detect perfusion alterations before clinical parameters change.

## Methods

### Study design

This study was approved by the local clinical research ethics committee (Decision Number 286 − 20) and registered at the German Clinical Trials Register (File Number DRKS00024048).

All procedures performed in this study were in accordance with the ethical standards of the 1964 Helsinki Declaration. Informed consent was obtained from all individual participants included in the study.

### Eligibility criteria

All participants were recruited in our clinic between December 2020 and February 2022. To be included in this study, patients had to be at least 18 years of age and in good general health (ASA I–II). All patients were Caucasian to ensure that the pigmentation of the gingiva would be comparable, although it was shown in prior LDF studies that skin pigmentation does not affect perfusion measurements significantly [21, 22].

Based on previous studies [23–25], gingivitis was defined by the presence of strong bleeding on probing at the interdental papilla (mean PBI > 2). Having a history of periodontitis was no exclusion criterion since our perfusion measurements took place at the interdental papilla with a measurement depth of just 1 mm. Nevertheless, patients who had signs of an acute infection or intraoral swelling were excluded. Other exclusion criteria covered: patients who smoked less than two hours before the examination, patients with missing teeth or dental implants, and patients with diabetes or other diseases that affect peripheral vascular perfusion.

### Sample size calculation

The existing literature on clinical trials of gingival perfusion methods was reviewed to calculate a sensible sample size range.

In particular, the required sample size was derived based on a study by Rodriguez-Martinez [26], which included 60 patients (12 without and 48 with gingivitis) and showed a correlation between probing depth, the GI, and the gingival perfusion index using LDF alone.

Furthermore, a study by Barry et al. [20], who used the same LDF-TS device with a different probe head in a single-arm study with 42 healthy patients, was taken into account for sample size calculation.

Since LDF-TS is a relatively new method to evaluate gingival perfusion, the basal flow differences were defined as the primary outcome to calculate the sample size.

The statistical program G* Power Version 3.1.9.6 (Heinrich-Heine-Universität, Düsseldorf, Germany) was used with an alpha value of 0.05, an effect size of 0.6, and a statistical power of 80%. Based on these parameters, a sample size of 20 patients was determined to reject the null hypothesis concerning primary basal flow differences with 80% power and a 95% confidence interval. Based on the studies mentioned above and other benchmark studies [27, 28], a sample size of at least 50 patients with and 50 patients without clinically manifest gingivitis was determined to be a sufficient. Hence, patients were recruited until both groups included at least 50 patients.

### Measurement procedure

All the measurements started with the use of the LDF-TS, and all patients were measured and examined by the same physician.

**Fig. 1:**
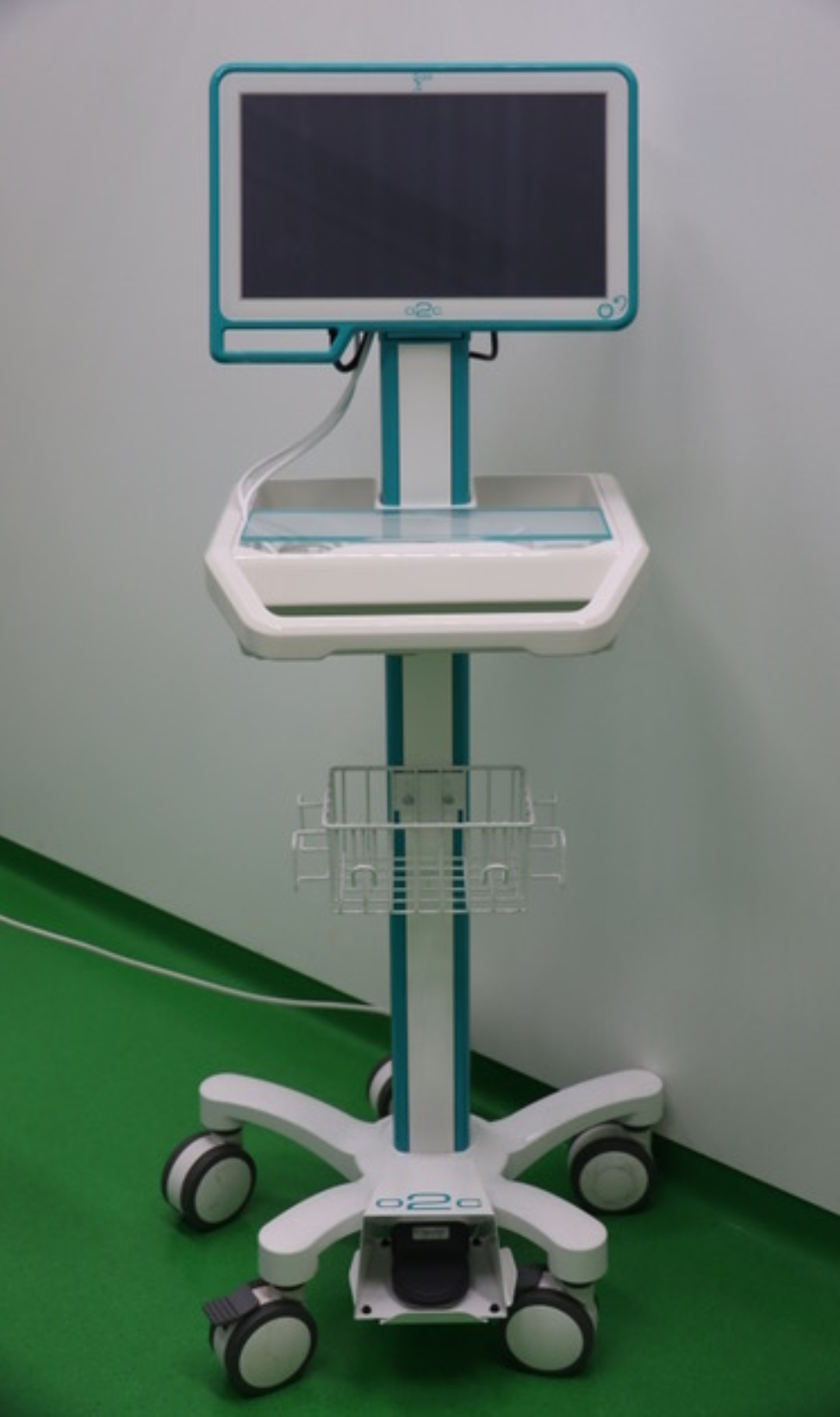
The “oxygen to see” (O2C) device

The O2C device is capable of collecting perfusion data on oxygen saturation SO_2_ (%), the relative amount of hemoglobin rHb (AU), and blood flow (AU; Fig. 1), making it a commonly used device in microsurgery [17–19, 29]. Depending on the choice of the probe, intraoral use is also possible [20].

Laser Doppler flowmetry and tissue spectrophotometry (LDF-TS), which is used in the “oxygen to see” (O2C) device (LEA-Medizintechnik, Gießen, Germany), is a common method in maxillo-facial and plastic surgery.

**Fig. 2:**
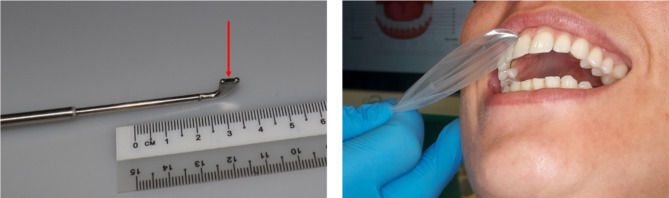
Gingival probe head of the “oxygen to see” (O2C) device

In this study, each patient was lying in a comfortable position, and the light above the dental chair was switched off to ensure an optimal setting for the O2C device measurement without any interference. All measurements used the LSX-41 gingival probe (LEA-Medizintechnik, Gießen, Germany) with a 5 × 2 mm dimension and a predefined measurement depth of 1 mm. The probe head in our study is specifically manufactured for gingival measurements, with two small batches that can be placed on adjacent teeth for constant stability, with the sensor and laser between them (Fig. 2). This ensures undisturbed measurement without noteworthy compression of the papilla.

**Fig. 3:**
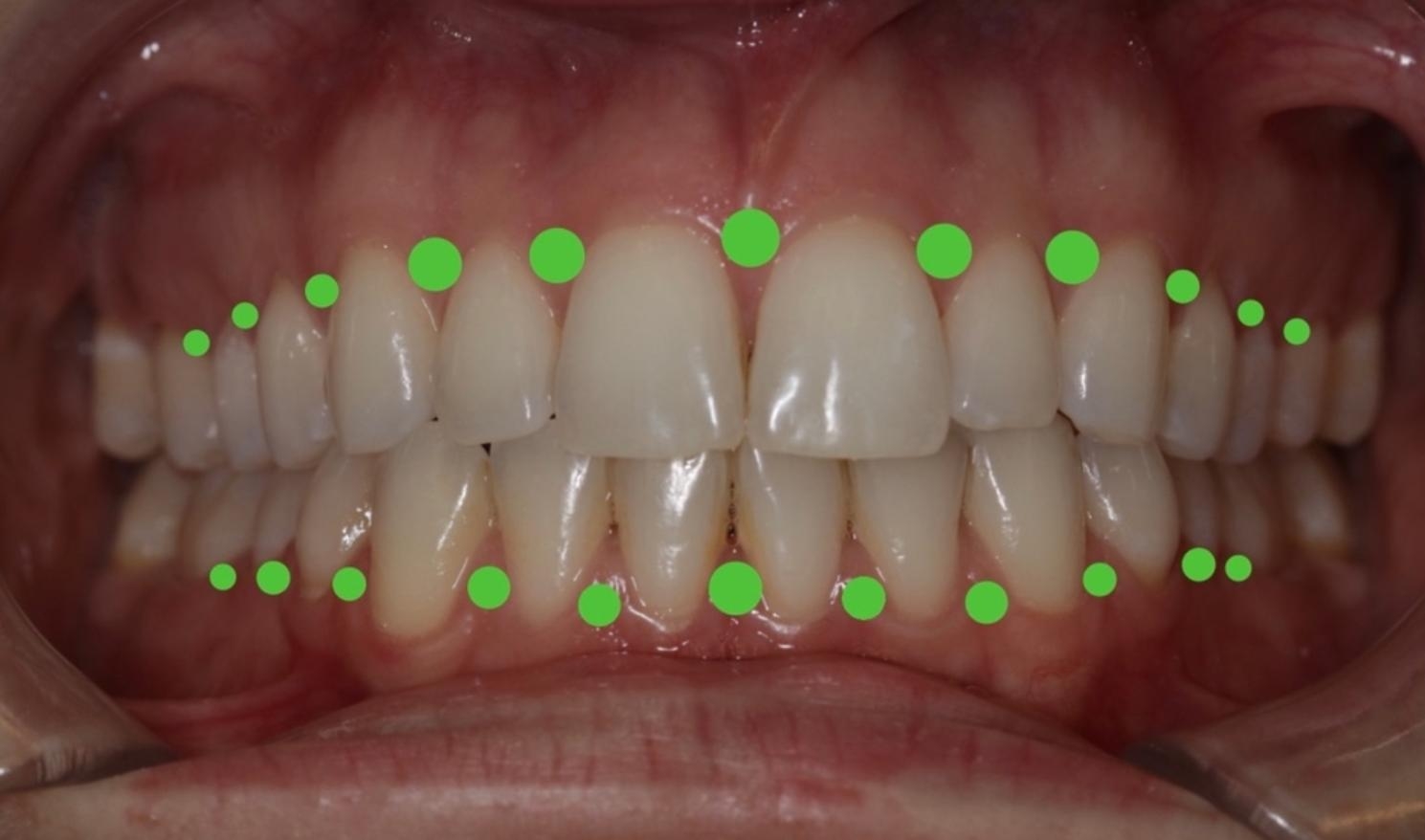
Measurement scheme of 22 points in the maxilla and mandible

22 papillae were measured per patient to gather an adequate dataset of individual patient perfusion. Measurements were first taken at the upper right jaw at the papilla between the first molar and the second premolar and then continued at the upper left jaw, the lower left jaw, and subsequently, the lower right jaw (Fig. 3).

At the start of each perfusion measurement period, the examiner had five seconds to adjust the fit of the probe head before measurements were recorded for the duration of ten seconds thereafter. This visual control mechanism ensured that the examiner was aware of his micro-movements.

If the patient or examiner moved unexpectedly, each measurement could be repeated if necessary.

### Clinical classification

After the perfusion measurements were completed, the patients were clinically examined. The examiner used a conventional periodontal probe with millimeter marks for all measurements.

The clinical periodontal examination included interproximal probing depth measurements (distance from the gingival margin to sulcus bottom), bleeding records (PBI by Saxer and Mühlemann), plaque index, and gingival biotype.

The plaque index based on the classification system developed by Löe and Silness [10] was determined by the amount of plaque on the adjacent teeth (0 = no plaque; 1 = thin plaque layer at the gingival margin, only detectable by wiping the periodontal probe; 2 = moderate plaque layer at the gingiva margin, observable with the human eye, no interdental plaque; 3 = a lot of plaque at the gingival margin, interdental plaque), and a mean PBI value was calculated for the 22 papillae measured (first molar to first molar in the upper and lower jaw) [9].

Generalized gingivitis was defined by a mean PBI higher than 2 based on the findings of Norderyd et al. [23] and Trombelli et al. [24]. The mean PBI was calculated as the sum of all papillae measured (including the ones without bleeding) and then divided by the 22 measuring points. As the PBI classification considers the amount of bleeding, that parameter was chosen over the simple number of papillae which showed BOP for a more nuanced and clear classification for patients with a clinically manifest gingivitis.

Patients with mean PBI values of 0–2 (0 = no bleeding; 1 = a single bleeding point; 2 = several bleeding.

points or one small band of bleeding) were included in the healthy group. Patients with mean PBI values higher than 2 (3 = the interdental triangle fills with blood; 4 = heavy bleeding, blood flowing over the teeth and gingiva) were assigned to the gingivitis group.

Probing depths were measured at the same 22 papillae which were previously examined by LDF-TS. Each patient´s gingival biotype was identified based on the transparency of the periodontal probe through the gingival margin in the buccal sulcus. If the probe shined through the gingiva, the biotype was considered thin; if the probe was not visible, the biotype was registered as thick [30, 31].

After the perfusion and clinical measurements, the resulting datasets were combined, and correlations between the perfusion parameters and clinical findings were evaluated.

Our primary aim was to analyze if significant differences in perfusion parameters between patients with and without gingivitis were observable, if there was a significant difference between points with and without BOP, and between patients who smoked compared to non-smokers. Beyond this, we checked for a difference between perfusion parameters between the sexes and the maxilla and mandible.

### Statistical analysis

For categorical data (sex, smoking habits, biotype, plaque index), differences between groups were analyzed using chi-square tests. Metric data (age, sex, mean probing depth, mean oxygen saturation, mean relative amount of hemoglobin rHb, mean blood flow and mean number of bleeding papillae) were evaluated using Mann–Whitney tests, as they lacked a Gaussian distribution according to the Shapiro- Wilk test.

Testing for differences in mean blood flow between groups, we controlled for sex, age, biotype, and smoking habits using multivariate regression analysis. Receiver operating characteristics (ROCs) were calculated, and the Youden Index was used to determine the theoretically optimal cut-off value of mean blood flow for predicting gingivitis [32]. P-values at a threshold of < 0.05 were considered to be significant.

## Results

Our study contains a total of 114 patients (53 male, 61 female), including 64 without gingivitis (26 male, 38 female) and 50 with gingivitis (27 male, 23 female). The mean age of the patients without gingivitis was 31.61 years (SD ± 13.31), while the mean age of those with gingivitis was 33.82 years (SD ± 15.35). There were no statistical differences in the sex (p = 0.155) or age distributions (p = 0.839) between groups.

The study sample included 33 patients with regular smoking habits, of whom 12 were assigned to the non-gingivitis group, and 21 were assigned to the gingivitis group. However, all patients confirmed that they had not smoked for at least two hours before the examination. Smoking was observed significantly more often in the gingivitis group (P = 0.007) but had no significant impact on the perfusion parameters measured (gingivitis vs. non-gingivitis patients: mean SO2: p = 0.207, mean rHb: p = 0.724 and mean flow: p = 0.171) and there was also no significant difference in the plaque indices of smokers and non-smokers in our study (p = 0.111).

Of all the patients, 74 had a thick gingival biotype (38 without and 36 with gingivitis), and 40 exhibited a thin biotype (26 without and 14 with gingivitis). The gingiva-type distributions did not differ between groups (p = 0.161) (Table 1).

**Table 1:** Patient collective and group characteristics

	Non-gingivitis group	Gingivitis group	Total	p- value
**Sex**				
Male	26	27	53	0.155
Female	38	23	61	
**Mean age**				
	31.61 (SD ± 13.31)	33.82 (SD ± 15.35)	32.60 (SD ± 14.22)	0.839
**Smoking habits**				
Yes	12	21	33	**0.007**
No	52	29	81	
**Gingival biotype**				
thick	38	36	74	0.161
thin	26	14	40	
**Plaque index**				
0	49	5	54	**< 0.001**
1	14	18	32	
2	1	14	15	
3	0	13	13	
**Prevalence of mean probing depths of patients showing < or ≥ 2 mm**				
< 2 mm	44	12	56	**< 0.001**
≥ 2 mm	20	38	58	
**Mean probing depths**				
	1.89 (SD ± 0.54)	2.44 (SD ± 0.99)	2.13 (SD ± 0.82)	**< 0.001**
**Mean number of bleeding papillae**				
	4.13 (SD ± 0.39)	11.22 (SD ± 4.98)	7.24 (SD ± 5.63)	**< 0.001**

Legend: Distribution of sex, smoking habits, gingival biotype, plaque index, and prevalence of mean probing depths is shown in absolute numbers. For categorical data (sex, smoking habits, biotype, plaque index, the prevalence of mean probing depths of patients showing < or ≥ 2 mm), differences between groups were analyzed using a chi-square test. Metric data (age, mean probing depth, mean number of bleeding papillae) was evaluated using a Mann–Whitney test

Patients who were assigned to the gingivitis group exhibited significantly higher values in plaque index values than patients in the non-gingivitis group (p < 0.001) and also showed significantly more often mean probing depths **≥** 2 mm (p < 0.001).

**Fig. 4:**
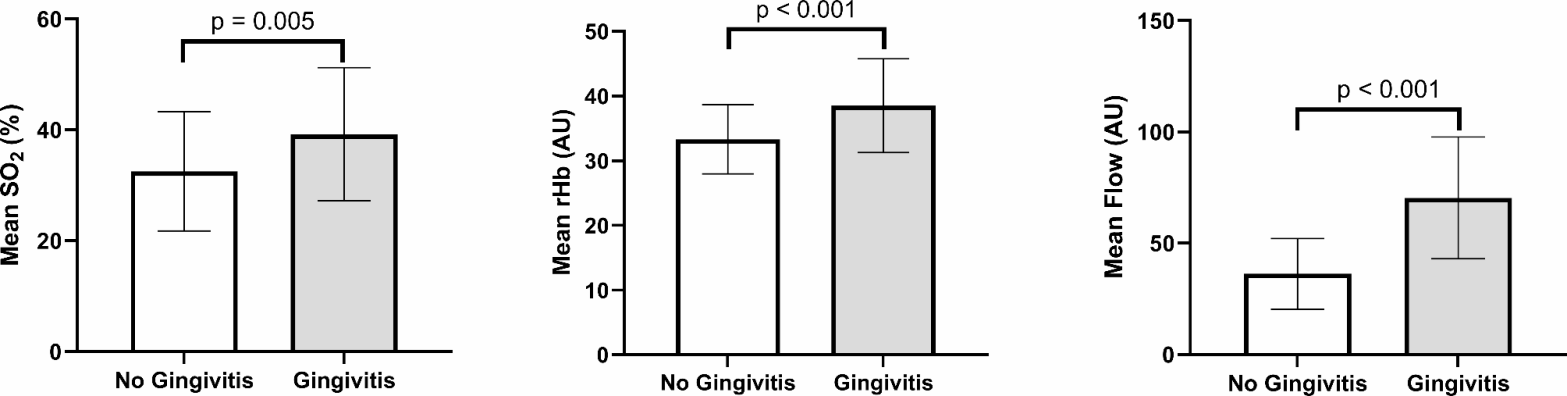
Mean oxygen saturation, the relative amount of hemoglobin, and flow values compared between patients with and without gingivitis Bars for mean values (SO_2_, rHb, and Flow) with whiskers for SD

The mean oxygen saturation SO2 (%), the mean relative amount of hemoglobin rHb (AU), and mean blood flow (AU) all differed significantly between the groups with and without gingivitis (p = 0.005, p < 0.001, and p < 0.001, respectively) (Fig. 4).

When controlling for sex (p = 0.114), age (p = 0.939), gingival biotype (p = 0.876), and smoking habits (p = 0.119) through regression analysis, the difference in mean blood flow values between the gingivitis and non-gingivitis patients remained significant (p < 0.001) (Table 2).

The optimal cut-off value of mean blood flow for predicting gingivitis was determined to be at > 40 AU (AUC = 0.878; p < 0.001; CI 0.817–0.939; sensitivity 0.90, specificity 0.67).

**Table 2:** Regression analysis was performed to test the differences in mean flow in arbitrary units (AU) between patients with and without gingivitis

Parameter	Value	ß	P- value
Gingivitis	No vs. Yes	0.633	**< 0.001**
Sex	Male vs. Female	-0.121	0.144
Age	(In years)	0.006	0.939
Gingival biotype	Think vs. Thin	-0.013	0.876
Smoking habits	Non-smokers vs. Smokers	-0.123	0.119

**Fig. 5:**
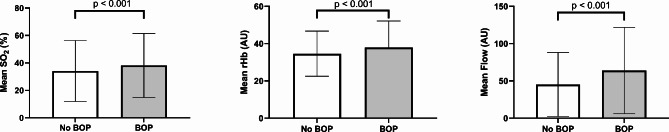
Oxygen saturation, the relative amount of hemoglobin and flow values compared between points with and without BOP Bars for mean values (SO_2_, rHb, and Flow) with whiskers for SD

When comparing the single points measured concerning BOP, all three parameters are significantly higher at the points featuring BOP (p < 0.001; Fig. 5).

**Fig. 6:**
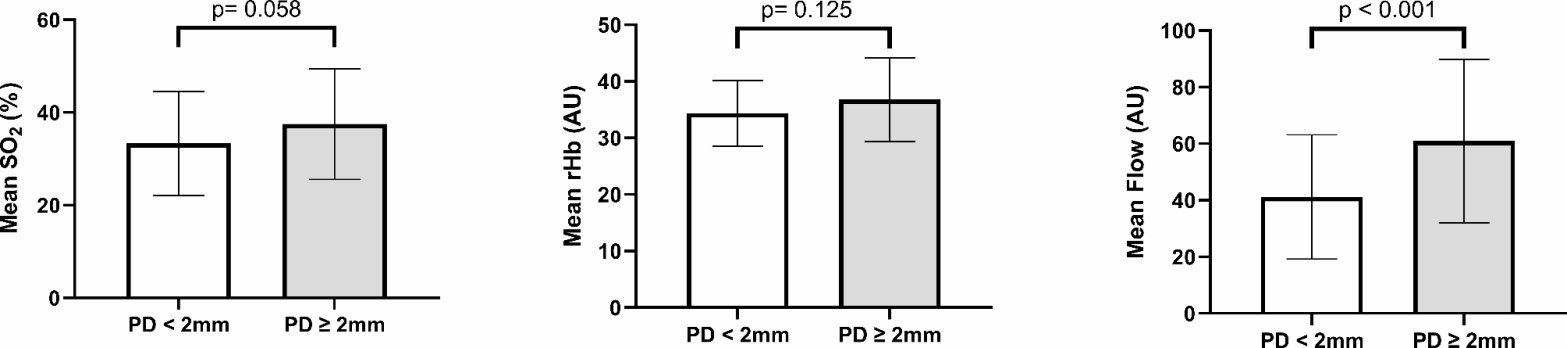
Mean oxygen saturation, the relative amount of hemoglobin, and flow values compared between patients with mean probing depths < or ≥ 2 mm Bars for mean values (SO_2_, rHb, and Flow) with whiskers for SD

Mean probing depths ≥ 2 mm also showed a significantly higher mean blood flow (p < 0.001) but no significant differences in mean oxygen saturation SO2 (p = 0.058) or the mean relative amount of hemoglobin rHb (p = 0.125) (Fig. 6).

**Fig. 7:**
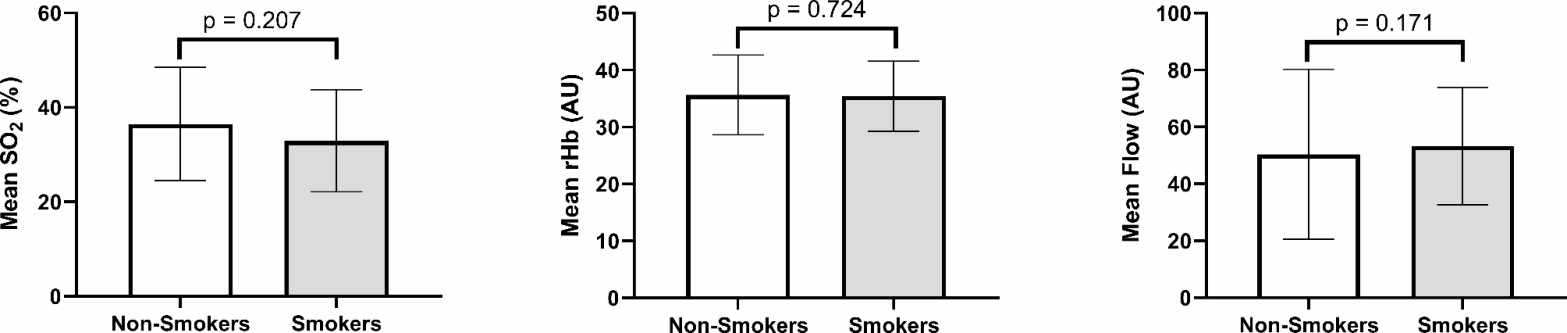
Mean oxygen saturation, the relative amount of hemoglobin, and flow values compared between smokers and non-smokers Bars for mean values (SO_2_, rHb, and Flow) with whiskers for SD

When comparing the perfusion values between smokers and non-smokers, mean oxygen saturation (p = 0.207), mean relative amount of hemoglobin (p = 0.724), and mean blood flow (p = 0.171) did not differ significantly (Fig. 7).

Mean oxygen saturation SO2 (%) and mean blood flow (AU) were not significantly different between men and women in either group (p = 0.085 and p = 0.088, respectively). However, the mean relative amount of hemoglobin rHb (AU) was significantly different between the sexes in favor of men (p < 0.001; Supplementary Figure S1).

Moreover, both groups displayed significant differences in oxygen saturation (p < 0.009), the mean relative amount of hemoglobin rHb (p > 0.001), and mean blood flow (p < 0.001), with the mandible featuring higher values than the maxilla (Supplementary Figure S2).

## Discussion

Using the relatively novel method of LDF-TS, significant differences were found in mean oxygen saturation, relative amount of hemoglobin, and flow values between patients with and without gingivitis.

Therefore, this diagnostic tool is considered to be a promising addition clinical examinations, which can detect typical signs of gingival inflammation, such as bleeding on probing, edema, enlarged pocket depths, and high plaque levels.

There are different approaches to classifying patients as gingivitis patients: Three common methods are the Gingiva Bleeding Index by Ainamo and Bay [33], the GI by Löe and Silness [10], and the PBI by Saxer and Mühlemann [9]. They all have a right to exist and highlight different clinical examination features.

We decided to base our mapping of patients into healthy and gingivitis patients on the PBI instead of BOP as a critical parameter as we wanted to compare relatively straightforward cases of heavy bleeding on probing with healthy patients with a focus on the perfusion parameters. In our opinion, the PBI classification is best suited to measure the amount of bleeding. However, there was a significant difference between the groups not only in the intensity of bleeding of probing measured by the PBI, but also in the number of bleeding papillae: Patients assigned to the gingivitis group showed significantly more bleeding sites than patients in the non-gingivitis group.

The gingivitis group showed mean BOP > 30% at the 22 measured sites, thus the group assignment is also in line with definitions of gingivitis based on the quantity of bleeding sites [34].

Likewise, there are also different options to grade the amount of plaque: O’Leary et al. assess the presence or absence of plaque [35], whereas Löe and Silness [10] weigh the amount of plaque in addition. In our study, the index developed by Löe and Silness was applied to indicate the relationship between manifest gingivitis and a high plaque level.

In our study population, patients with gingivitis showed significantly higher plaque index values than patients without gingivitis, which is in line with the findings of Breuer et al. [36].

As age and sex can be associated with a higher likelihood of gingivitis [37], our groups were tested for significant differences in these two parameters. The age and sex distributions of the two groups were not significantly different and were, therefore, comparable.

Patients with an acute infection or swelling were excluded, but we did not exclude patients with known periodontitis or a history of periodontal treatment.

Both mean probing depths as well as the prevalence of mean probing depths ≥ 2 mm, which were significantly greater in the gingivitis group than in the group with the healthy patients, show that even initial inflammatory processes can have an impact on attachment and marginal bone loss. This finding is in line with a multicenter study by Zimmermann et al., who found a positive correlation between BOP and increased probing depths [38].

Gaining knowledge about the blood flow, relative amount of hemoglobin, and oxygen saturation can help to explain the metabolism of gingival inflammation and to detect deeper underlying processes [20, 39, 40].

The LDF method can be used to examine gingival blood flow [41–44]. This method has been used in prior studies to evaluate differences between healthy and inflammatory gingival conditions [15, 45], as well as the impact of a new toothbrush on gingival perfusion [46], the effect of platelet-rich fibrin on gingival regeneration after tooth extraction [47], and the vascularization of free gingival grafts [27].

LDF-TS is a novel method, which is largely used to monitor microvascular transplants in plastic and maxillofacial surgery [16–19, 29], and the combination of all three parameters (blood flow, oxygen saturation, and relative amount of hemoglobin) indicates whether the examined tissue falls within a healthy metabolic range.

High flow values indicate a state of hyperemia caused by the inflammatory reaction, which causes higher blood flow and leads to clinical symptoms such as redness, swelling, and bleeding on probing [37, 48].

In line with prior studies by Matsuo et al., Kerdvongbundit et al., and Gleissner et al. [15, 28, 49], a significantly higher blood flow was found in our study in patients with clinical signs of gingivitis.

Furthermore, by performing a ROC analysis, it could be shown that blood flow values apply to the detection of gingivitis, as values over 40 AU were predictive for gingivitis with a sensitivity of 90%.

The mean SO_2_ value was significantly higher in patients with gingivitis than in the healthy group. As the O2C device measures postcapillary blood flow, a higher percentage of oxygen is a sign of lower oxygen consumption at the papilla; this can be a sign of hypoxia, which is common in damaged periodontal tissue [50, 51].

A high hemoglobin concentration can be a warning sign for degenerative vessel constrictions, and oxygen saturation can signal oxygen consumption and hypoxic conditions of tissues affected by periodontitis [50, 51].

Appropriately, the higher hemoglobin values we found in patients with gingivitis align with the theory that patients exhibit degenerative vessel structures at the interdental gingiva, which is the first to atrophy in patients with gingivitis [52].

Although the margins for gingival perfusion have not yet been clearly defined, in a study by Barry et al. [20] a modified LDF-TS probe for gingival measurements was used and perfusion parameters differed between the alveolar mucosa in the maxilla and mandible. In contrast to this study, the improvised LDF-TS probe used by Barry et al. had a greater diameter (5 × 5 mm) and measuring depth (3 mm). Furthermore, as the probe used by them was too big to be placed directly adjacent to the teeth, the measurements took place at the alveolar mucosa, palate, retromolar trigone, and lingual surface, whereas the measurements in our study were taken directly at the papilla with a 2 × 5-mm probe that measures at a depth of 1 mm; thus, the results of these studies are not directly comparable. The advantage of our small probe is that it is designed to be placed right upon the papilla and can detect the perfusion of the small capillaries in the papilla that run directly under the surface. As the alveolar gingiva and papilla are thin mucosal layers, the measuring depth of this study was much closer to the vascular processes [30].

Like Barry et al., as a secondary outcome, our study also found higher oxygen saturation and mean hemoglobin values in the mandible than in the maxilla. In contrast to the findings of Barry et al., significantly higher blood flow in the papillae of the lower jaw than in the papillae of the upper jaw was detected in our study. The explanation for this remains unclear; however, this finding could be explained by the inferior alveolar artery, which is mainly relevant to gingival perfusion in the mandible [53]. Since all the present measurements were performed on the papillae of the buccal side, blood flow from the palatal side was not registered, where the blood supply from the incisive and palatal foramina in the upper jaw originates.

Comparing our further secondary findings, Wang et al. [40] also found a difference in the perfusion parameters between men and women in that oxygen saturation was lower in male patients. In the present study, SO_2_ and flow measurements were not significantly different between the sexes, but hemoglobin values were significantly higher in men than in women. This finding falls in line with men having generally higher hemoglobin values than women [54].

Based on the aforementioned studies, we measured 22 papillae in the upper and lower jaws of 114 patients to obtain an adequate sample of 2508 papillae in total. By comparing the mean values of oxygen saturation, the amount of relative hemoglobin, and blood flow, it was ensured that intraindividual outliers did not overly impact the results and that it was possible to find margins in the mean perfusion parameters that differed between patients with and without gingivitis.

To avoid differences in measurements taken by different examiners, it was ensured that the same dentist performed the perfusion measurements and the clinical check-up.

Since our measurements were superficial (1 mm measurement depth) and took place at the interdental papilla, they were primarily able to detect marginal gingival hyperemia, rather than underlying periodontitis which takes place at the lower periodontium. Still, higher mean flow values for mean probing depths ≥ 2 mm were found. Since there is a smooth transition between chronic gingivitis and a beginning attachment loss in periodontitis, this underlines the importance of early detection of perfusion changes at the papilla.

Smoking can also decrease gum perfusion and mask underlying inflammation [1, 55]. In the present study, it was ensured that the patients did not smoke at least two hours before the examination to eliminate any short-term effects on perfusion [55–57].

Although there were significantly more smokers in the gingivitis group, neither mean SO_2_, mean rHb-, nor mean blood flow values or plaque index differed significantly when comparing smokers to non-smokers.

Furthermore, a regression analysis was performed to control for smoking habits, and the mean blood flow values remained significantly different between the non-gingivitis and gingivitis groups. This finding indicates that LDF-TS seems to be a diagnostic tool suitable for detecting gingival inflammation independent of smoking habits.

Gingivitis is common in patients of all ages and sexes [37]. The detection and treatment of gingivitis not only plays a vital role in the prevention of periodontal disease [48] but may also have an impact on general health [58].

In summary, microvascular processes– such as hypoxia, early vessel degeneration, and inflammatorily increased blood flow– can be detected reliably with the gingival probe of the O2C device and differ significantly between patients with clinically manifest gingivitis and healthy patients.

As this study was the first to use a specialized gingival probe with a small head and low measuring depth, future studies with this probe could be conducted to evaluate changes in papillary blood flow between different periodontal and peri-implant conditions, as well as how these conditions change and progress over time.

## Conclusion

As a non-invasive method, LDF-TS can help detect gingival hyperemia and enrich the clinical examination of the gum and its appearance. Furthermore, this diagnostic tool can be used to quantify the amount of perfusion, as it is one of the main characteristics of interdental inflammation. In particular, flow values above 40 AU are predictive of hyperemic processes in the papillae. The O2C device can be used to objectively evaluate perfusion parameters during routine examinations, after periodontal treatments, or to show the progression of hyperperfusion before any apparent changes in clinical parameters are observed. It can be useful to detect the early diagnosis of gingivitis, however this finding should be validated in future studies.

### Electronic supplementary material

Below is the link to the electronic supplementary material.

Supplementary Material 1

## Data Availability

The datasets used and/or analyzed during the current study are available from the corresponding author upon reasonable request.

## List of abbreviations

AU: Arbitrary units
AUC: Area under the curve
GI: Gingiva Index
LDF-TS: Laser Doppler flowmetry and tissue spectrophotometry
LDF: Laser Doppler flowmetry
O2C device: “oxygen to see” device.
PBI: Papilla Bleeding Index
rHb: Relative amount of hemoglobin
ROCs: Receiver operating characteristics
SO2: Oxygen saturation
