# Supplementary material for: Evaluation of perfusion parameters of gingival inflammation using laser Doppler flowmetry and tissue spectrophotometry– a prospective comparative clinical study
Source: BMC Oral Health. 2023 Oct 14;23:761. doi: 10.1186/s12903-023-03507-9 (PMC10576369; doi:10.1186/s12903-023-03507-9)
Supplement: Supplementary file 1 — Supplementary Material 1 [file 12903_2023_3507_MOESM1_ESM.pdf]

Supplementary material:

Supplementary Figure 1. Mean oxygen saturation, the relative amount of hemoglobin, and flow values compared between men and women

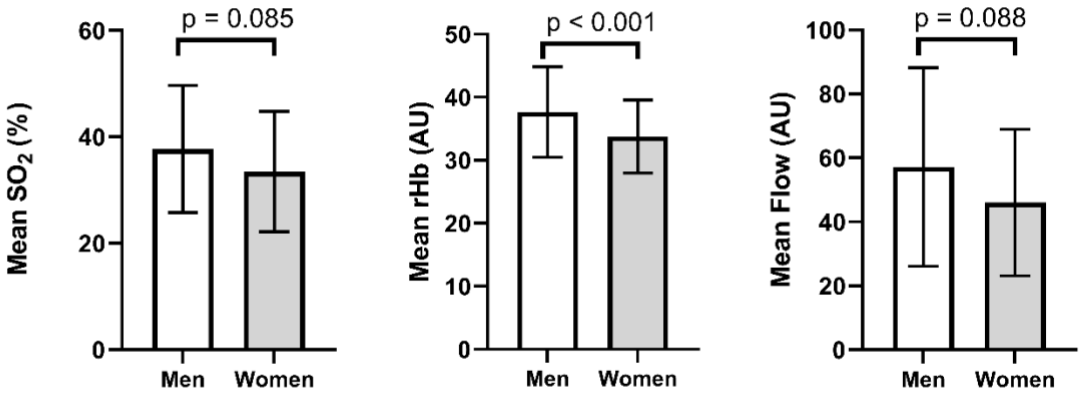

Bars for mean values (SO_2, rHb, and Flow) with whiskers for SD.

Mean oxygen saturation SO2 (%) and mean blood flow (AU) were not significantly different between men and women in either group ($p = 0.085$ and $p = 0.088$, respectively). However, the mean relative amount of hemoglobin rHb (AU) was significantly different between the sexes in favor of men ($p < 0.001$; Fig. 8, Supplementary Material).

Supplementary Figure 2. Mean oxygen saturation, the relative amount of hemoglobin, and flow values compared between the maxilla and the mandible

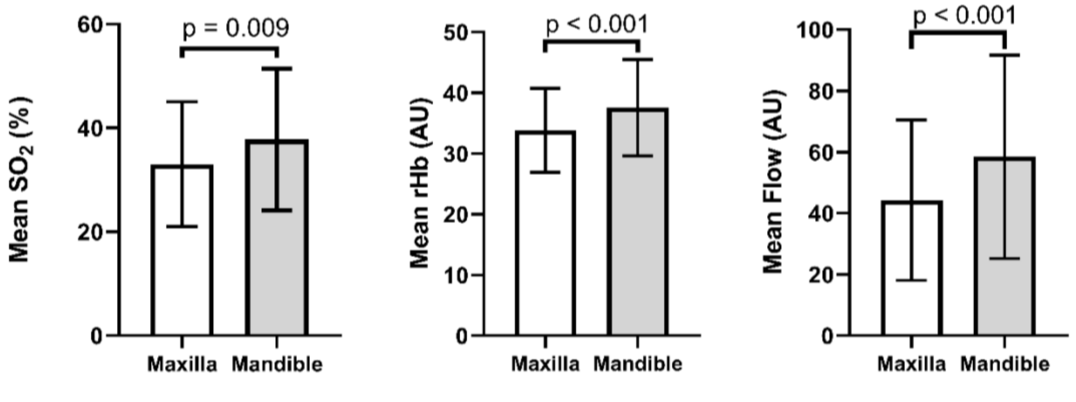

Bars for mean values (SO_2, rHb, and Flow) with whiskers for SD.

Moreover, both groups displayed significant differences in oxygen saturation ($p < 0.009$), the mean relative amount of hemoglobin rHb ($p > 0.001$), and mean blood flow ($p < 0.001$), with the mandible featuring higher values than the maxilla (Fig. 9, Supplementary Material).
